# Causality in Schwinger’s Picture of Quantum Mechanics

**DOI:** 10.3390/e24010075

**Published:** 2022-01-01

**Authors:** Florio M. Ciaglia, Fabio Di Cosmo, Alberto Ibort, Giuseppe Marmo, Luca Schiavone, Alessandro Zampini

**Affiliations:** 1 Departamento de Matemáticas, Universidad Carlos III de Madrid, Leganés, Avda. de la Universidad 30, 28911 Madrid, Spain; fciaglia@math.uc3m.es (F.M.C.); fcosmo@math.uc3m.es (F.D.C.); luca.schiavone@unina.it (L.S.); 2 Instituto de Ciencias Matemáticas (ICMAT), CSIC-UAM-UC3M-UCM, C. Nicolás Cabrera 13-15, 28049 Madrid, Spain; 3 Dipartimento di Scienze Fisiche “E. Pancini”, Università Federico II, Napoii, 40, 80138 Naples, Italy; marmo@na.infn.it; 4 Dipartimento di Matematica ed Applicazioni, Università Federico II, Napoli, 40, 80138 Naples, Italy; azampini@na.infn.it

**Keywords:** causality, groupoids, causal categories, causal sets, von Neumann algebras, incidence algebras, triangular algebras

## Abstract

This paper begins the study of the relation between causality and quantum mechanics, taking advantage of the groupoidal description of quantum mechanical systems inspired by Schwinger’s picture of quantum mechanics. After identifying causal structures on groupoids with a particular class of subcategories, called causal categories accordingly, it will be shown that causal structures can be recovered from a particular class of non-selfadjoint class of algebras, known as triangular operator algebras, contained in the von Neumann algebra of the groupoid of the quantum system. As a consequence of this, Sorkin’s incidence theorem will be proved and some illustrative examples will be discussed.

## 1. Introduction: Causal Structures vs. Quantum Mechanics

The principle of causation, ”cause precedes effect” or ”every effect has a cause”, is the bedrock of modern science, and beyond it, is magic. In fact, *“we can assert that scientific research, especially as it has been developed after the Renaissance, can be considered to be primarily the practical application of the principle of causation based on observation, analysis (deductive or inductive), experiment, formation of hypothesis and the formulation of theories and models”* [1]. The scientific basis for the principle of causation itself was loosely implemented in the interaction description of physical laws (Newtonian physics first and Einstein’s general relativity thereafter since the beginning of the 20th century). The peculiar role played by ”time” in quantum mechanics, and the difficulties inherent to the foundations of quantum field theories, has left aside the analysis of the principle of causation itself in quantum theories.

This paper addresses the problem of the role of causality in quantum mechanics, taking advantage of the recent developments on the categorical/groupoidal description of quantum systems inspired by Schwinger’s picture of quantum mechanics (see, for instance, [2,3,4,5,6,7,8,9,10,11] and references therein).

Causality is often described in a geometrical setting by means of a Lorentzian metric on a manifold of spacetime events. Specifically, the family of events that can be “causally” related is identified with a subset of points on a smooth manifold M, four dimensional in most physical applications, whose geometrical properties are encoded in a metric tensor $\eta =\eta_{\mu \nu }dx^{\mu }dx^{\nu }$, of signature −+⋯+, satisfying some additional properties that make them adequate for physical interpretation (see discussion below in Section 2).

This geometrical approach to causality drinks directly from Riemann’s conceptualization of geometrical structures, it is firmly rooted in physical grounds by Einstein’s description of the gravitational field, and has been used ever since to provide the basic background for physical theories. In particular, it was found quite early that major modifications to the so-called standard Copenhagen interpretation were needed to render quantum mechanics consistent with this description of causality. After strenuous work, these modifications led to today’s various approaches to “quantum field theories”.

However, this is not the only route leading to the implementation of the principle of causation in physical theories. Already, Kronheimer and Penrose [12] considered an abstract description of the physical interpretation of the causality relation between the events of the manifold M: “*An event x causally precedes an event y if the interpretative principles would allow an occurrence at x to influence what happens at y,..., such models contain a second, more restrictive relation of chronological precedence, corresponding to the possible time-ordering of events on the world-line of an idealized observer, whose velocity is less than that of light. In certain manifolds such analysis of causal and chronological precedence may become ambiguous or impossible; but we shall treat the existence of consistent relations of this character as a criterion of admissibility*”.

Kronheimer and Penrose proceeded to treat these notions of causal and chronological precedence on an axiomatic basis, describing the event-space M as a set equipped with two order-like relations: *causality* (⪯), and *chronology* (≪), satisfying appropriate axioms (see Section 2 below for details). The systems of axioms should be kept as small and physically reasonable as possible without pretending to reproduce the manifold approach in its entirety. In actuality, one of the main aims of such analysis would be [12] “*to admit structures which can be very different from a manifold. The possibility arises, for example, of a locally countable or discrete event-space equipped with causal relations macroscopically similar to those of a space-time continuum*”. Certainly, these ideas were exploited and expanded upon by R. Sorkin’s causal sets program [13], where a variation of Kronheimer and Penrose axioms was established as the fundamental causal structure of a physical theory of gravity (see, for instance, [14] and references therein).

It is hard to emphasize further the impact that the geometric implementation of causality and its derived arguments, such as relativistic covariance and locality, had on the development of quantum mechanics and quantum field theory. In actuality, it was precisely trying to build a theory incorporating locality and relativistic covariance that led R. Haag to build a consistent axiomatic approach to quantum field theory known today as algebraic quantum field theory (AQFT) [15,16]. This approach is based on the notion of Haag–Kastler nets, i.e., an assignment of a $C^{*}$-algebra $A(O_{y,x})$ to any causal double-cone $O_{y,x}=\langle y,x\rangle$ in a spacetime M, satisfying a set of axioms inspired by the notions of ”locality” and “relativistic covariance”. Some of the main contributions that the development of AQFT has provided are a solid framework to the theory of superselection rules, the CPT theorem, and the spin–statistics connection.

Given the importance of causal structures highlighted by the previous discussion, it appears at least reasonable to ask whether there is a way to understand how said causal structures emerge from more fundamental quantum mechanical principles. However, in this respect, it is relevant to note that, at least in principle, it may appear as paradoxical that specific causal structures, such as Minkowski spacetime with its standard future time orientation, could provide the background for a theory that is time reversible, as quantum mechanics is (in accordance with Feynman’s principle of microscopic reversibility [17,18]). Therefore, the purpose of this work is to try to shed some light on the meaning of this question by combining the axiomatic approach to causality in the tradition of Kronheimer, Penrose, Sorkin, etc., and the recently developed formulation of quantum theories based on the groupoidal formulation of quantum mechanics inspired by Schwinger’s seminal work.

The high level of abstraction involved in the groupoidal picture of quantum mechanics makes it well adapted to discuss these issues. Indeed, there is a relevant stream of investigative efforts which focuses on considering the foundational aspects of quantum mechanics on a more abstract level as a way to better understand their nature (see, for instance, the recent works [19,20,21,22,23,24,25,26,27]).

In the groupoidal picture of quantum mechanical systems, a groupoid is associated with each family of experimental settings used to describe the system (see later, Section 3.2, and [2,3,4,5,6,7,8,9,10] for details). The objects *x* of the groupoid are the outcomes of the measurements performed on the system, and its arrows, or morphisms, are the physical transitions that the system experiences. Each transition, say α:x→y, has an intrinsic orientation, the outcome *x* being the source and *y* the target of the observed transition. The fundamental microscopic reversibility principle stated forcefully by Feynman is implemented as a main axiom of the theory by imposing the family of transitions to form a groupoid, that is, to be such that, for each transition α:x→y, there is an inverse transition $\alpha^{-1}:y\to x$, whose composition with α leaves the system unchanged. Therefore, at the kinematical level, there is no preferred “time orientation” or “arrow of time”. Specific causal relations among events, and the outcomes of the theory associated with them, emerge only when we introduce comparison dynamics in the system, that is, an auxiliary system whose dynamical evolution is well known and that serves us to account for our observations (for instance, a clock carried by the observer), and to exchange this information with other observers and their own experimental settings. It is well known that natural conditions on the consistency of such comparison dynamics leads to the determination of the possible kinematical invariance groups of the theory, that, under a few simple assumptions, turn out to be the Poincaré and Galilei groups (see, for instance, [28,29,30]).

In this context, it just makes sense that the outputs of a (sub)system of the given system can be used to describe the “arrow of time” of the system. Note that these outputs (the ticking of a clock, for instance) are part of the groupoidal description of the system, together with other outputs used/needed by the experimenter to “locate” or “individuate” the system (think, for instance, of the experimental setting used to study the behavior of an electron in a cavity or box).

The germ of this idea was elevated to a principle by A. Connes and C. Rovelli introducing the notion of a thermodynamic time in the description of quantum systems in a general covariant setting, the so called Connes–Rovelli thermodynamic time hypothesis [31]. More precisely, such hypothesis considers that the system is described by a certain von Neumann algebra of observables and that the dynamics provided by the Tomita–Takesaki modular flow associated to a given reference state provides the natural choice for an arrow of time.

In this paper, we work around this idea by providing an algebraic characterization of causality relations which is suitable to describe in terms of the von Neumann algebra of the given system, which, in turn, is provided by the von Neumann algebra of the groupoid associated with it. Quite obviously, we will work in the framework of the recently introduced groupoidal description of quantum systems. In this setting, causal structures will be identified with a particular class of subcategories of the groupoid of the system under investigation. This formalism will lead us to identify causal relations with a particular instance of subalgebras of the von Neumann algebra of the quantum system. Specifically, these subalgebras are not ∗-algebras but, rather, triangular operator algebras, that is, a subclass of the family of algebras known as Kadison–Singer algebras [32,33]. It will be shown how to reconstruct the causal relation in the space of outcomes of the groupoid from the algebras involved. This reconstruction theorem constitutes a nontrivial extension of Sorkin’s theorem stating the existence of a one-to-one correspondence between maximal indecomposable ideals in the incidence algebra of the given causal set and the events in this set that reproduces the causal relation (see Section 3, Theorem 1). Thus, the main conclusion of this paper is that Schwinger’s picture of quantum mechanics in its modern presentation based on groupoids and their algebras provides a new way to deal with causality in physical theories.

An important observation regarding the full program is that, in order to incorporate both the mathematical technical tools and the physical background ideas, it is necessary to extend the theory of causal relations from its standard topological/differentiable setting to a measure theoretical one. These aspects are discussed in detail in Section 3 of this paper. However, in order to avoid technical difficulties and to help the main ideas be more easily apprehended, only the discrete situation will be discussed in the analytical part of this work, Section 4. Thus, after revising the standard geometrical approach to causality in Section 2, Section 3 is devoted to the construction of the relevant categorical notions associated with the notion of a causal structure and, finally, in Section 4, the operator algebras associated with categorical causal relations will be studied in relation with the von Neumann algebra of the given quantum system, and the previously mentioned reconstruction theorem will be proved.

## 2. The Geometric Theory of Causality

As it was already mentioned in the introduction, A. Einstein laid a solid background for the formal description of physical causal relations as a consequence of his critical analysis of the structure of space and time. For the purposes of this work, Einstein’s main observation can be stated as the identification of physical events with points in a smooth manifold M carrying a Lorenztian metric η, of which Minkowski spacetime is the simplest possible realization. More precisely, a (geometric) spacetime (M,η) is a time-oriented connected Lorentz manifold. A Lorentz manifold is a smooth manifold (here, manifolds are always assumed to be Hausdorff and paracompact). M of dimension *m* (typically m=4), endowed with a nondegenerate metric tensor η of signature (−,+,⋯,+).

Causality emerges from the metric structure of spacetime because of its associated distribution of light cones. Specifically, each tangent space $T_{x}M$ contains a causal cone $C\left(x\right)=\{v\in T_{x}M|\eta \left(v,v\right)=0,v\neq 0\}$, that decomposes in two connected components, called the causal cones at *x*. A time-orientation on M consists of a smooth choice of one of the two causal cones at every x∈M, which will be called the future cone and denoted by $C_{+}\left(x\right)$ (and the nonchosen one will be called the past cone, denoted $C_{-}\left(x\right)$). Note that the smoothness condition before amounts to the family of cones $C={⋃}_{x\in M}C\left(x\right)\subset TM$ being a smooth submanifold of TM. This submanifold is invariant under the free action of the $Z_{2}$ group given by inversion v↦−v. The quotient space $C/Z_{2}$ is a manifold and we will say that the Lorentzian manifold is time-orientable if there exists a smooth section of the canonical projection $C\to C/Z_{2}$. This is equivalent to saying that there is a smooth choice of a causal cone $C^{+}\left(x\right)$, for every x∈M.

A tangent vector v∈TM is timelike, null, causal, or spacelike, if η(v,v)<0, η(v,v)=0, η(v,v)≤0, η(v,v)>0, respectively (the conventions used here differ slightly from the usual ones in standard Lorentzian causality theory [34] as ‘causal’ usually refers to vectors such that η(v,v)≤0, v≠0, but we will adopt the previous terminology as it makes simpler the matching with more abstract notions of causality). The definitions above extend naturally to vector fields *X* on M and curves γ:I→M. More explicitly, let *I* denote an interval [a,b]⊂R, (−∞≤a<b≤+∞), a timelike, lightlike, or causal curve is a piecewise smooth curve γ:I→M, such that not only the tangent vectors $\dot{\gamma }\left(s\right)$, s∈I, are timelike, lightlike or causal, respectively, but also the two lateral tangent vectors at each break-point must lie in the same causal cone. It is easily shown that a Lorentzian manifold is time-orientable if and only if (iff) it admits a globally defined timelike vector field *T* (which can be chosen to be complete). Such vector field *T* can be chosen to be future-oriented at all points x∈M, i.e., $T\left(x\right)\in C^{+}\left(x\right)$, and then, a causal tangent vector *v* is future oriented iff $\eta_{x}\left(v,T\left(x\right)\right)<0$. Any Lorentzian manifold admits a time-orientable double covering. Any Riemannian metric *g* on a smooth manifold gives rise to a time-orientable Lorentzian manifold provided that we choose a nonvanishing vector field *X* (the can be chosen to be normalized, g(X,X)=1) by means of η=g−2g(X)⊗g(X), with g(X) the canonical 1-form associated with *X*, hence the existence of time-oriented Lorentzian structures reduces to the determination of nonvanishing vector fields [35] (Prop. 5.37).

Following Kronheimer and Penrose’s notion of causal relation, and complementing the description introduced in the introduction, we can state [12] that *“An event x precedes an event y if a message could be transmitted from x to y”*, or even more, we can consider the following information-based definition of causality: x⪯y if information can be transmitted from *x* to *y*. Because information is physical and transmission implies that a physical channel between *x* and *y* can be created such that information would be carried though it, we conclude that the causal relation x⪯y amounts to the existence of a causal curve γ:I=[a,b]→M, such that γ(a)=x and γ(b)=y, which constitutes the geometrical implementation of the causal relation ⪯. We will also write γ:x→y.

We will define the causal and timelike future domains $J^{+}\left(x\right)$, $I^{+}\left(x\right)$ as the sets of events in M that can be reached by causal and timelike curves, respectively. In a similar way, the causal and timelike past domains $J^{-}\left(x\right)$, $I^{-}\left(x\right)$ are defined. The timelike domains $I^{+}\left(x\right)$ are open sets and the open double cones $<y,x>=I^{-}\left(y\right)\cap I^{+}\left(x\right)$ generate a topology on M called the Alexandrov topology. A spacetime (M,η) is called strongly causal if the Alexandrov topology coincides with the standard manifold topology. Strongly causal spaces lie in the middle of the causality ladder (see, for instance, [34,36] and references therein), and exhibit a wealth of significant geometrical properties, among them the fact that their spaces of light rays are smooth manifolds, provided that an additional technical condition is satisfied (see, for instance, [37] and references therein).

Now, Kronheimer and Penrose’s axioms for causal spaces can be spelled out from the geometrical properties of (M,η), that is, we consider three different relations ≪, ⪯ and →, called, respectively, chronological, causal, and horismos, and defined as x≪y if $y\in I^{+}\left(x\right)$, x⪯y if $y\in J^{+}\left(x\right)$, and x→y, if x⪯y but x≪/y, respectively. Then, the relation ⪯ is a partial order, i.e., it is reflexive, transitive, and antisymmetric; the chronological relation ≪ is irreflexive, transitive, and antisymmetric and, in addition, it satisfies that x≪y⇒x⪯y, x⪯y≪z⇒x≪z, and x≪y⪯z⇒x≪z (these properties can be succinctly expressed saying that ≺ is a partial order and ≪ is irreflexive contained in ≺).

Clearly, the geometrical theory of causality depicted so far is appropriate until we need to consider situations where there is no natural geometrical spacetime background, as it happens, for instance, when dealing with many problems in quantum mechanics or in attempting to understand quantum properties of the spacetime itself. Kronheimer and Penrose’s idea [12] of an abstract description of causality, following the remarks in the previous paragraphs, fits naturally into these situations and was taken back by Rafael Sorkin as a departing point for a fresh approach to quantum gravity [38].

## 3. Algebraic Causality: A Categorical Approach

### 3.1. Borel Causal Sets

Sorkin’s notion of causal sets (or “causets”), that is, discrete countable sets Ω with a partial order ⪯, such that the intervals [y,x]={z∈Ω∣x⪯z⪯} are finite [14], implies the assumption that the basic notion underneath a causal structure on a physical system is a set of events Ω partially ordered by a relation of causal precedence. Causal sets provide a natural background to understand basic questions on gravity. A relevant step in this program is achieved by a theorem that reproduces Gelfand’s duality theorem for Abelian $C^{*}$-algebras: a compact space Ω is naturally homeomorphic to the space of maximal ideals of the $C^{*}$-algebra of its continuous functions. Sorkin’s theorem asserts that a (finite) causal set can be recovered from the structure of a family of ideals in its incidence algebra (see [39] for a recent proof of this theorem). A new proof of this theorem will be obtained as a consequence of the results discussed in Section 4.

**Theorem** **1.** *[40] Let (Ω,⪯) be a finite causal set. There is a one-to-one correspondence between maximal indecomposable ideals of the incidence algebra ν(Ω,⪯) and points in* Ω*. Moreover, x⪯y iff $J_{y}J_{x}\neq 0$, where $J_{x}$ is the maximal indecomposable ideal canonically associated with x∈Ω.*

Therefore, the abstract, axiomatic approach to causality is a natural way to learn more about the role of causality in physical theories. In fact, such abstract axiomatic approach can be placed nicely in the algebraic setting of category theory. Before proceeding to do so, first we will enrich the notion of causal order as a partial order relation on sets with a measurable structure that, even if it is not going to be the central theme of this work, is the natural framework to set the general analysis. The reason for that is that causal sets are assumed to satisfy an interval finiteness condition [14], a condition which is not satisfied in many natural applications. However, it is often the case in most physical applications that the space of events Ω carries a measurable structure. Thus, it will be assumed that Ω carries a measurable structure given by a σ-algebra B of sets on Ω, typically the algebra of all subsets of Ω when Ω is discrete countable.

A measurable structure B on Ω is a family of sets, including the empty set and Ω itself, which is closed with respect to countable intersections and complements (hence with respect to countable unions too). The physical interpretation of such family of sets is that sets Δ∈B correspond to actual “events” on M, i.e., outcomes of actual measurements or observations performed on the system. The axiom concerning countable intersections of sets $\Delta_{n}$ in B corresponds to an idealization of the actual measurement processes taking place, that is, it reflects the possibility of repeating an observation an unlimited number of times, and the complementary axiom reflects that if Δ∈B, then Ω∖Δ∈B, is just the negation of the observation Δ. An additional requirement, the consequence of such idealization, is that “atomic events” {x}, x∈Ω, are measurable sets (Formally, it can be assumed that for any *x* there exists a family $\left\{\Delta_{n}\left(x\right)\right\}$) of measurable sets such that $\cap_{n}\Delta_{n}\left(x\right)=\left\{x\right\}$. A space Ω equipped with a measurable structure B satisfying the previous conditions will also be called a Borel space and the elements Δ∈B Borel sets (Borel sets are often referred, more restrictively, as the sets in the σ-algebra generated by a given topology on Ω). This constitutes a crude axiomatic setting for a proper algebraic description of measurement processes as described, for instance, by Resende using the notion of quantals in the context of topology in “pointless” spaces [24,25], even though using Borel structures is sufficient for the situations that will be met in this work.

A map $F:\left(\Omega ,B\right)\to \left(\Omega^{\prime },B^{\prime }\right)$ will be said to be measurable if $F^{-1}\left(\Delta \right)\in B$, for any $\Delta \in B^{\prime }$, and we will say that two Borel spaces (Ω,B) and $\left(\Omega^{\prime },B^{\prime }\right)$ are Borel isomorphic if there is an invertible measurable map $F:\Omega \to \Omega^{\prime }$ such that $F^{-1}$ is measurable too.

Then, we will say that the causal structure determined by the partial order ⪯ on the Borel space (Ω,B) is a standard causal structure if Ω is a standard Borel space (i.e., Borel isomorphic to a Borel subspace of a separable complete metrizable topological space), and ⪯ is consistent with such measurable structure, that is, if we denote by R⊂Ω×Ω the graph of the relation defined by ⪯, R={(y,x)∈Ω×Ω∣x⪯y}, then R is a Borel subset of Ω×Ω, hence R is a standard Borel space itself. Standard Borel spaces have very good properties from the point of view of measure theory. They are Borel isomorphic either to a countable or finite set (our main situation in the current work), or to the interval [0,1] with its standard Borel structure. It would be necessary in many applications to consider a more general class of measurable spaces though (see, for instance, Proposition 1 below), called analytic spaces, which are spaces Borel isomorphic to continuous images of Polish spaces (separable, complete metrizable topological spaces). We will keep our attention on standard Borel spaces for the rest of this section and, in the coming ones, we will restrict our interest to countable discrete spaces with their natural Borel structure.

Given a partial order relation R⊂Ω×Ω, we will denote by ∘ the natural composition map $\circ :R^{\left(2\right)}\to R$, given by

(z,y)∘(y,x)=(z,x),x,y,z∈Ω,(z,y),(y,x)∈R,

where $R^{\left(2\right)}$ denotes the subset of R×R determined by consistent pairs, i.e, (y,x) is consistent with (v,u) if y=u.

We denote by s,t the maps from R to Ω defined as s(y,x)=x and t(y,x)=x, called, respectively, the “source” or “past” map, and the “target” or “future” map of the causal relation R. Note that if R is a Borel set, then s,t are measurable maps (indeed, if Δ⊂Ω is measurable, then $s^{-1}\left(\Delta \right)=R\cap \left(\Omega \times \Delta \right)$ is the intersection of two measurable sets, hence it is measurable too). In most applications, the past and future maps s,t, are measurable submersions, that is, they are subjective, measurable, and the image of a measurable set is measurable. Note that such condition is satisfied if both s,t, possess measurable right inverses. The previous discussion can be summarized as a formal definition:

**Definition** **1.** *A Borel causal set structure on a standard Borel space (Ω,B) is a partial-order relation ⪯ such that its associated relation R⊂Ω×Ω is a Borel subset of Ω×Ω, the canonical composition ∘ is a Borel map, and the source and target maps are Borel submersions. The space Ω together with its Borel structure B and a Borel causal relation ⪯ will be called a causal Borel space (or just causal space if there is no risk of confusion).*

Note that, if R is a Borel causal structure on Ω, the canonical map i:Ω→R, given by i(x)=(x,x), is Borel because $i^{-1}\left(\Delta \right)=\Delta_{\Omega }\cap \Delta$, where $\Delta_{\Omega }\subset \Omega \times \Omega$ denotes the diagonal subset, which is Borel.

Given two events x,y∈Ω, x⪯y, the double cone defined by x,y is the set of events *z* such that x⪯z⪯y and it will be denoted as [y,x]. We will denote by <y,x> the “open” double cone of events such that x≺z≺y, where x≺y means that x⪯y and x≠y. Each double cone (open or closed) in a causal space inherits a natural causal Borel structure. Given a causal space Ω, there is a natural topology associated with it, its Alexandrov topology, which is the topology generated by the family of open double cones <y,x>.

Given two causal Borel spaces $\left(\Omega_{a},B_{a},{⪯}_{a}\right)$, a=1,2, a Borel map $\phi :\Omega_{1}\to \Omega_{2}$ will be called causal if $\phi \left(x\right){⪯}_{2}\phi \left(y\right)$ whenever $x{⪯}_{1}y$, with $x,y\in \Omega_{1}$. Two causal Borel spaces will be said to be causal isomorphic if there is a causal Borel isomorphism between them.

Simple, natural examples of causal Borel spaces include directed graphs, Kronheimer–Penrose causal structures, and geometrical spacetimes. Thus, the simplest examples of Borel causal structures are provided by oriented acyclic graphs Γ, that is, consider a set Ω of vertices and a (at most countable) set Γ of links, i.e., a collection of ordered pairs (y,x), x,y∈Ω. We consider that the graph defined in this way is acyclic, that is, it possesses no closed paths. Then, let $R_{\Gamma }$ be the smallest partial order relation on Ω containing Γ. In other words, consider the smallest subset $R_{\Gamma }\subset \Omega \times \Omega$ satisfying the axioms of a partial order containing Γ. We may call $R_{\Gamma }$ the partial order generated by the graph Γ. Then, $R_{\Gamma }$ defines a Borel causal structure with respect to the Borel structure generated by the vertices {x}, x∈Ω.

The simplest examples of such causal spaces are provided by any subset *C* of the set of integer numbers, Z, with its natural total order: n⪯m iff m−n≥0. Note that, with the previous notations, open and closed double cones in Z are related as <m,n>=[m−1,n+1]. A causal space will be said to carry a linear order if it is causal isomorphic to <a,b>, a,b∈Z (where a,b could be ±∞). Sorkin’s causal sets are particular instances of the previous examples [13,41].

As it was discussed at the end of Section 2, Kronheimer and Penrose’s causal spaces are quadruples (M,⪯,≪,→) where the three relations ⪯, ≪, and →, satisfy the axioms [12] (KR axioms):

1. ⪯ is a partial order.
2. ≪ is areflexive, i.e., not x≪x.
3. ≪ is finer that ⪯, that is, if x≪y, then x⪯y; x≪y⪯z→x≪z; x⪯y≪z→x<<z.
4. x→y iff x⪯y and not x≪y;

and they provide good examples of Borel causal set structures. In fact, if (M,⪯) is a complete metrizable separable topological space whose topology is generated by the Alexandrov topology defined by open cones <y,x>, satisfying KP axioms, then the chronological order ⪯ determines a standard Borel causal space relation $R_{⪯}$ on M.

A most relevant class of standard causal structures is provided by geometric causal relations associated with metric structures on spacetimes (see Section 2). Thus, if (M,η) is a strongly causal spacetime, then it is a standard Borel space with respect to the Borel structure defined by the Alexandrov topology. A large class of spacetimes (M,η) of physical interest satisfy this requirement, most notably the so-called globally hyperbolic spacetimes [36]. A particular instance of such spaces is given by the standard Minkowski space M in dimension *m*, which is diffeomorphic to $R^{m}$ equipped with the metric $\eta =-{\left(dx^{0}\right)}^{2}+{\left(dx^{1}\right)}^{2}+\cdots +{\left(dx^{m-1}\right)}^{2}$. In general, the causal order relation ⪯ defined on a spacetime (M,η) will not be standard, but analytic, as mentioned above. In fact, we have the following proposition:

**Proposition** **1.** *The natural partial order relation* ⪯ *defined on a strongly causal spacetime (M,η) determines an analytic causal Borel structure on M.*

**Proof.**  The Borel structure will be that generated by the topology of the manifold. Because the space is strongly causal, this implies that the topology and the Borel structure are both generated by double cones. Hence, to show that the causal structure defined by x⪯y iff $y\in J^{+}\left(x\right)$ is Borel, it suffices to show that the graph of the relation R={(y,x)∈M×M∣x⪯y} is an analytic Borel set in M×M. For that, it suffices to show that R is the image under a continuous map of a Borel set. Consider for each n∈N, the set $P_{n}\left(M\right)$, the space of *n*-polygonal causal geodesics on M, that is, $\gamma \in P_{n}\left(M\right)$ is the union of *n* causal geodesics $\gamma_{l}:\left[a_{l},b_{l}\right]\to M$ such that $a_{l+1}=b_{l}$. Any two causally related events x⪯y can be joined by an *n*-polygonal causal geodesic for *n* large enough. Hence, the image of the continuous map $F:{⋃}_{n=0}^{\infty }P_{n}\left(M\right)\to M\times M$, given by $F\left(\gamma \right)=(\gamma \left(b_{n}\right),\gamma \left(a_{1}\right))$, is R. □

### 3.2. The Categorical Approach to Causality: Causal Structures as Borel Categories

We will now discuss a way to identify causal structures with abstract algebraic notions, more precisely with categories, which will be particularly useful for the purposes of the present work (see also [27,42,43] for other discussions of causality in the categorical setting). The relation R has the structure of a category whose composition law is given by (z,y)∘(y,x)=(z,x), whenever x⪯y⪯z. The composition law ∘ reflects the transitive property of the causal relation ⪯; moreover, the units $1_{x}$ of the category R are the diagonal pairs (x,x), and the antisymmetric property reflects the fact that only units have inverses.

On the other hand, any subcategory R of the groupoid of pairs P(Ω)=Ω×Ω defines a partial order in Ω setting x⪯y iff (y,x)∈R, provided that $R\cap R^{-1}=i\left(\Omega \right)$, where $R^{-1}=\left\{\left(y,x\right)|\left(x,y\right)\in R\right\}$, and i:Ω→R is the map sending the object *x* to the unit $1_{x}=\left(x,x\right)$. We will say that a subcategory R of the pair groupoid P(Ω) of a measurable set Ω such that $R\cap R^{-1}=i\left(\Omega \right)$ is measurable if the partial order defined by it determines a measurable causal structure on Ω. We will also denote the category R as R⇉Ω, to emphasize the role played by the past and future maps s,t, s(y,x)=x, t(y,x)=y, and we will say that R⇉Ω is a measurable causal category. A subset S⊂Ω×Ω, also called a quiver over Ω, will be said to generate the category R if R is the smallest subcategory in P(Ω) containing S. All this lead us to the following formal definition:

**Definition** **2.** *A Borel category is a small category C⇉Ω carrying a Borel structure such that the source, target, and composition maps are Borel. In addition, it will be assumed that the space of units* Ω *is a Borel subset of C and the source and target maps are Borel submersions.*

As it is customary, given a morphism α in the category C⇉Ω, we will denote it as α:x→y, where x=s(α) is the source of α and y=t(β) is the target of α. The composition law in C will be denoted as β∘α and is defined provided that t(α)=s(β), the family of such composable pairs is denoted as $C^{\left(2\right)}$. The units of C will be denoted as $1_{x}$, for any object x∈Ω. Then, we will denote by *i* the canonical assignment $x\mapsto 1_{x}$. We will also denote by $C^{\mathrm{opp}}$ the opposite category to C, i.e., the category whose arrows are the inverses of the arrows in C.

Using the previous notions, it can be said that a Borel causal set structure on the standard Borel space Ω is a Borel category R contained in Ω×Ω, i.e., a Borel subcategory of the groupoid of pairs P(Ω). On the other hand, if C⇉Ω is a Borel category over Ω, then the canonical map Π:C→P(Ω)=Ω×Ω, given by

Π(α)=(t×s)(α)=(t(α),s(α))=(y,x),α:x→y∈C,

determines a relation on Ω. This relation R=Π(C) will be a partial order if $\Pi \left(C\cap C^{\mathrm{opp}}\right)=\Delta_{\Omega }$, with $\Delta_{\Omega }=\left\{\left(x,x\right)|x\in \Omega \right\}$, the diagonal set of P(Ω). In particular, if $C\cap C^{\mathrm{opp}}=i\left(\Omega \right)$, then C induces a partial order on Ω given by x⪯y iff there exists α:x→y∈C. We will refer to this partial order as the partial order associated with the category C, and we will say that C⇉Ω is a causal category.

**Definition** **3.** *A causal Borel category is a Borel category C⇉Ω such that $\Pi \left(C\cap C^{\mathrm{opp}}\right)\subset \Delta_{\Omega }=i\left(\Omega \right)$. The induced partial order* ⪯ *on the Borel space* Ω*, defined as x⪯y iff there exists α:x→y in C, defines a Borel causal structure on* Ω *called the causal structure associated with C. The causal Borel category C will be said to be strict if $C\cap C^{\mathrm{opp}}=i\left(\Omega \right)$.*

We will upgrade the notations and terminology introduced in the previous sections to the current situation. We will denote by $C_{x}$ and $C^{y}$ the preimages $s^{-1}\left(x\right)$, x∈Ω, and $t^{-1}\left(y\right)$, y∈Ω, respectively. Note that, if C⇉Ω is a causal Borel category, the family of sets $C_{x}$ ( $C^{y}$) define a measurable partition of C.

As we did in Section 2, given x∈Ω, we will denote by $J^{+}\left(x\right)$ the causal future set of *x*, that is, $J^{+}\left(x\right)=\left\{y\in \Omega |x⪯y\right\}=\left\{y\in \Omega |\exists \alpha :x\to y\in C\right\}$. Note that $J^{+}\left(x\right)=t\left(s^{-1}\left(x\right)\right)=t\left(C_{x}\right)$, hence, it is a measurable set. Similarly, we define the past causal set $J^{-}\left(y\right)=\left\{x\in \Omega |x⪯y\right\}=\left\{x\in \Omega |\exists \alpha :x\to y\in C\right\}$. We will denote by [y,x] the causal interval defined by x,y, that is, $\left[y,x\right]=\left\{z\in \Omega |x⪯z⪯y\right\}=J^{+}\left(x\right)\cap J^{-}\left(y\right)$. Causal intervals are measurable sets and they are also termed (closed) “double cones”. We will say that the causal structure is past separating if $J^{-}\left(y\right)=J^{-}\left(y^{\prime }\right)$ implies that $y=y^{\prime }$ (future separating is defined in a similar way).

It will be relevant for the discussion to follow to consider the analogue of the notion of transitivity for causal categories. Any (causal) category C⇉Ω generates a groupoid G⇉M, which is the smallest groupoid containing C. Given an object x∈Ω, the orbit of *x*, with respect to the groupoid G, is the set of all objects *y* such that there exists α:x→y∈G. The groupoid G is said to be transitive (or connected) if there is an *x* such that its orbit is the full set Ω. Clearly, a similar notion can be introduced for the category C where the notion of orbit is replaced by the causal sets $J^{+}\left(x\right)$ and $J^{-}\left(y\right)$. Then, we will say that the causal structure determined by the causal category C⇉Ω is future (past) transitive if there is x∈Ω such that $J^{+}\left(x\right)=\Omega$ ($J^{-}\left(x\right))=\Omega$, respectively). However, this notion of transitivity is too restrictive for the purposes of this research, as not even Minkowski space satisfies it. It is more natural to consider a weaker notion of transitivity, that will be referred in what follows as relative transitivity, defined as follows: the causal category C⇉Ω will be said to be future relative transitive, if for any x,y∈Ω, if there exists a finite sequence $z_{n}\in \Omega$ such that $z_{1}⪯x$, $z_{1}⪯z_{2}$, $z_{3}⪯z_{2}$, $z_{3}⪯z_{4},\ldots z_{n}⪯y$, in other words, $x,\;y\in J^{+}\left(\left\{z_{k}\right\}\right)$, with $J^{+}\left(U\right)=\left\{y\in \Omega |\exists x\in U,\alpha :x\to y\in C\right\}$. Similarly, past relative transitivity will be defined, and C will be said to be relative transitive if it is both past and future relative transitive.

**Figure 1:**
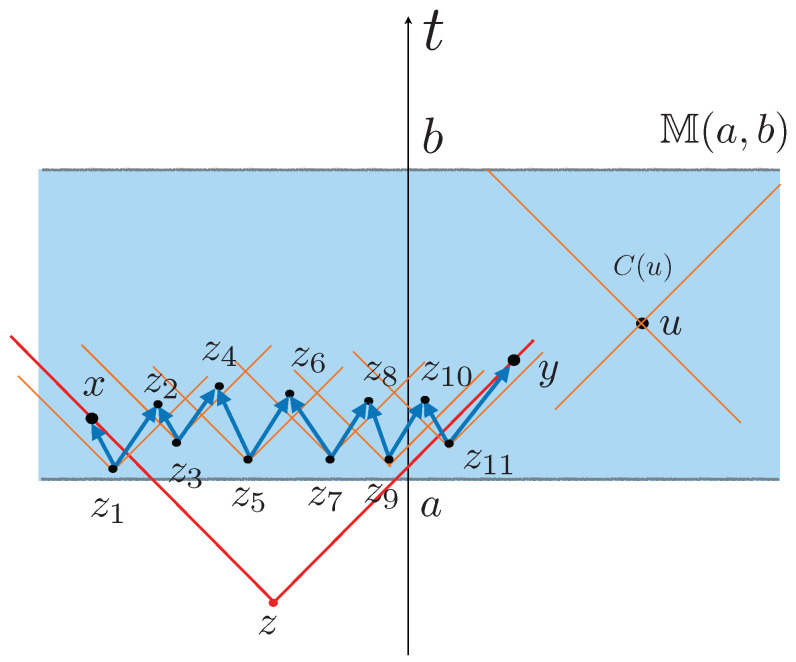
Diagram representing a Minkowski strip space M(a,b) (in blue) as a subspace of Minkowski space. The causal cone C(u) of an event *u* is marked in orange (right). Two events x,y not causally related can be joined by a seesaw path $\left(x,\;z_{1},\;z_{2},\;z_{3},\;z_{4},\;z_{5},\;z_{6},\;z_{7},\;z_{8},\;z_{9},\;z_{10},\;z_{11},\;y\right)$, consisting of causal geodesics (dark blue) contained in M(a, b). Note that the set of points $J^{-}\left(x\right)\cap J^{-}\left(y\right)$ in the common causal past of x, y, is the causal past $J^{-}\left(z\right)$ of *z* (in red) which is out of the Minkowski strip.

Clearly Minkowski causal category is relative transitive, and the notion even applies to Minkowski strips. Specifically, consider the strip in Minkowski space $M_{\left(a,b\right)}=\left\{x\in M|a<x^{0}<b\right\}$. Then, the strip $M_{\left(a,b\right)}$ with the causal structure inherited from M is relative transitive because any pair of events x,y, even if they are close to the boundary of $M_{\left(a,b\right)}$, can be joined by a “seesaw” path of causal relations (see Figure 1).

We conclude this section by introducing the notion of causal structure in a groupoid as a categorical causal structure contained in the groupoid itself.

**Definition** **4.** *Let G⇉Ω be a Borel groupoid. Then, a Borel causal structure on G is a subcategory C⊂G which is a Borel causal category over* Ω*. We will say that the causal structure C is transitive if $C\vee C^{-1}=G$, where $C\vee C^{-1}$ denotes the category generated by C and $C^{-1}$ where the subcategory $C^{-1}$ is identified with the opposite category ${C\;}^{\mathrm{opp}}$.*

As it was indicated before, Borel causal set relations are Borel causal structures on the groupoid of pairs P(Ω). If Γ is a discrete countable group, then a causal structure on it (considered itself as a category) will be a submonoid C⊂Γ, such that $C\cap C^{-1}=\left\{e\right\}$. We may consider, for instance, the causal structure on GL(n,C) defined by the set *C* of upper triangular matrices with unit diagonal. This example provides the name for the algebras discussed in the following section.

## 4. Analytic Causality: Groupoids and Quantum Mechanics

As it was discussed in the previous section, the categorification of the notion of a causal relation leads to the notion of a causal structure on a groupoid. Groupoids have proved to be the natural way to implement Schwinger’s notion of algebra of selective measurements, his proposal for a foundational description of quantum mechanical systems. Hence, the previous notions allows us to introduce a notion of causal structure on quantum systems.

As discussed in the introduction, the physical interpretation of a groupoid G⇉Ω is that morphisms α:x→y in the groupoid represent physical transitions of the system. Objects x,y∈Ω, represent outputs of measurements of physical observables. The partial associative composition law β∘α:x→z, of two transitions α:x→y, β:y→z, represent the observed concatenation of both (see, for instance, [2,3,7,11] for details). Hence, the notion of groupoid contains a notion of “precedence” implicitly encoded in its composition law, albeit symmetric, that is, Feynman’s microscopic reversibility principle imposes that any transition α:x→y, must posses an inverse $\alpha^{-1}:y\to x$, such that $\alpha^{-1}\circ \alpha =1_{x}$, and $\alpha \circ \alpha^{-1}=1_{y}$, in other words, the transition $\alpha^{-1}$ reverses the previously observed transition α, leaving the system unchanged. If a “clock” were part of the experimental setting (as it is customary), i.e., a “time” parameter is also part of the outcomes of the system, then, following the previous argument, transitions taking us back in time are also part of the groupoidal description of the system. The way to reconcile this with the perception of a causal structure on the description of quantum systems is by considering the “clock” as a classical system with its own independent (classical) dynamics.

The notions introduced in Section 3 allow for a different, more natural and physically deeper, way of understanding the emergence of causal structures on quantum systems by selecting a causal structure in the sense of Definition 4. Thus, instead of claiming that there is an “external” classical system (the “clock”) measuring the time of the observations performed on our quantum system, we will consider that there is a causal subcategory of the groupoid that has been selected to describe the given quantum system.

In this framework, the standard description of the “time” parameter in quantum mechanics would correspond to consider two separate systems described, respectively, by groupoids $G_{A}⇉\Omega_{A}$ and $G_{B}⇉\Omega_{B}$, of which $G_{A}$ is our quantum system of interest and $G_{B}$ describes a classical system with a prescribed dynamics (the “clock”). In such a case, the space of outputs $\Omega_{B}$ would be an interval $[t_{0},\;t_{1}]\subset R$ (or a interval in Z). The groupoid $G_{B}$, being a classical system, will be identified with the set $\Omega_{B}$ itself as no nontrivial transitions $\alpha_{B}:t\to s$ exist on $G_{B}$ apart from the units $1_{t}:t\to t$ (see [44] for details on dynamics of classical and quantum systems). Hence, the direct product of both systems $G_{A}\times \Omega_{B}⇉\Omega_{A}\times \Omega_{B}$ will represent the standard composition of both systems whose transitions will have the form $\left(\alpha ,1_{t}\right):\left(x,\;t\right)\to \left(y,t\right)$, so there is not an intrinsic notion of causal precedence build in the system. The causal relation on the space of outcomes (x,t) is introduced instead by hand as (x, t)⪯(y, s) iff t≤s (which is not a partial order relation as it is not antisymmetric because all transitions (α,t) occur “instantaneously”, i.e., at a fixed time *t*). This is clearly unphysical, and to mend it we have to introduce a relativistic picture, that eventually lead us to the intrinsic difficulties of quantum field theories. Because of all this, we conclude that a different approach must be considered to address the “problem of time” in quantum mechanics. The framework proposed in this work will help to clarify it, as will be shown in subsequent articles.

Instead of addressing the problem of time in quantum mechanics further, we will turn our attention to study the structure and properties of causal structures in the analytical context provided by the von Neumann algebra associated with a given groupoid. In doing so, we will be able to study them using their “incidence algebras”, that is, the abstract algebra that captures the relations between events. This algebra is much better understood from the perspective of the algebra of a Borel category as a subalgebra of the von Neumann algebra of a given groupoid. This would allow us to prove a reconstruction theorem that will encompass, among other things, Sorkin’s theorem, Theorem 1.

### 4.1. The Incidence Algebra of a Causal Relation and Sorkin’s Theorem

As was mentioned before, we will restrict ourselves in what follows to discuss the discrete case in order to keep the technical difficulties at bay. Thus, given a discrete countable Borel category C⇉Ω, equipped with the counting measure, we will denote by $L^{2}\left(C\right)$ the space of square summable functions on C, i.e., functions ψ:C→C, such that $\sum_{\alpha \in C}{\left|\psi \left(\alpha \right)\right|}^{2}<\infty$.

We will denote by C[C] the complex linear space generated by C, that is, the set of formal finite linear combinations $a=\sum_{\alpha \in C}a_{\alpha }\alpha$, with only a finite number of nonvanishing coefficients $a_{\alpha }\in C$. The linear space C[C] carries a canonical associative product defined as

$$b\cdot a=\sum_{\left(\beta ,\alpha \right)\in C^{\left(2\right)}}b_{\beta }a_{\alpha }\;\;\beta \circ \alpha \;,$$

and C[C] becomes an associative algebra (nonunital if C is infinite). Notice that C[C] is not a ∗-algebra because the natural ∗-operator $a\mapsto a^{*}=\sum_{\alpha }{\bar{a}}_{\alpha }\alpha^{-1}$, is not defined as, in general, $\alpha^{-1}\notin C$.

The algebra (C[C],·) is called the (algebraic) incidence algebra of C (actually, this is the standard terminology when restricting ourselves to the situation C[C]⊂P(Ω), i.e., C is a relation on Ω). However, in order to build a robust analytical framework, it is necessary to equip this algebra with a Banach algebra structure. There are two ways of doing this. The first one mimics the definition of the von Neumann algebra of a group(oid) and departs from the observation that the algebra C[C] carries a natural representation, its right regular representation in the space of bounded operators on $L^{2}\left(C\right)$, $R:C\left[C\right]\to B(L^{2}\left(C\right))$, defined as

(1) $$\left(R\left(a\right)\psi \right)\left(\beta \right)=\sum_{\left(\beta ,\alpha \right)\in C^{\left(2\right)}}a_{\alpha }\psi \left(\beta \circ \alpha \right)\;.$$

It is a simple computation to check that R(a·b)=R(a)R(b). We will define the (analytical) incidence algebra of the category C, and we denote it as ν(C), as the double commutant of the range of the regular representation *R*, that is,

$$\nu \left(C\right)=R{\left(C\left[C\right]\right)}^{\prime \prime }\;,$$

where $R{\left(C\left[C\right]\right)}^{\prime }=\left\{A\in B\left(L^{2}\left(C\right)\right)|\left[R\left(a\right),A\right]=0,\forall a\in C\left[C\right]\right\}$, and $R{\left(C\left[C\right]\right)}^{\prime \prime }={\left(R{\left(C\left[C\right]\right)}^{\prime }\right)}^{\prime }$. It is clear that the identity operator *I* belongs to ν(C) and is the unital element of ν(C). The algebra ν(C) inherits a Banach algebra structure from the operator norm on $B(L^{2}\left(C\right))$; however, we will delay the study of its structural properties until the discussion of the second way to construct it.

Ideals of incidence algebras play a fundamental role in the statement of Sorkin’s theorem. Let us recall that a left ideal J⊲A of an associative algebra is a subspace of A which is a left-A-module, i.e., such that AJ⊂J. Right ideals are defined in a similar way, and two-sided ideal, or just ideals, are both left and right ideals. Given two ideals J,I, we can define its sum, J+I, which is the ideal generated by the union of both. We will say that an ideal J is indecomposable if there are no two (nontrivial) ideals $J^{\prime }$, $J^{\prime \prime }$ such that $J=J^{\prime }+J^{\prime \prime }$. The product JI of two ideals is the ideal consisting of finite linear combinations $\sum_{i}x_{i}y_{i}$ of products of elements $x_{i}\in J$, $y_{i}\in I$ (note that, in general, JI⊂J∩I). With this terminology, an improved version of Sorkin’s theorem, Theorem 1, would be stated as follows:

**Theorem** **2.** *Let C⇉Ω be a discrete countable strict causal category. There is a one-to-one correspondence between events x∈Ω and maximal indecomposable ideals $J_{x}$ in the incidence algebra ν(C). Moreover, x⪯y, i.e., there is α:x→y∈C iff $J_{y}\cdot J_{x}\neq 0$.*

The second construction of ν(C) takes advantage of the situation when the category C is contained in a groupoid G, which is the situation we are mostly concerned with in this paper and that will be kept until the end. Thus, we will assume that a discrete countable groupoid G⇉Ω describing a certain quantum system is given and we will consider a causal structure on it, that is, a Borel causal subcategory C⊂G.

The groupoid G carries natural left and right representations on the space of bounded operators on $L^{2}\left(G\right)$. We will concentrate on the right regular representation $R:C\left[G\right]\to B(L^{2}\left(G\right))$, defined by a formula similar to Equation (1), that allows us to define the von Neumann algebra ν(G) of the groupoid as the double commutant $R{\left(C\left[G\right]\right)}^{\prime \prime }$, where C[G] is the ∗-algebra defined, as in the case of a category, by formal finite linear combinations of transitions α:x→y∈G. Note that the space C[G] can be identified naturally with the space of functions on G with compact support $C_{c}\left(G\right)$ with the convolution product $\left(f⋆g\right)\left(\alpha \right)=\sum_{\gamma \circ \beta =\alpha }f\left(\gamma \right)g\left(\beta \right)=\sum_{s(\beta )=s(\alpha )}f\left(\alpha \circ \beta^{-1}\right)g\left(\beta \right)=\sum_{t(\gamma )=t(\alpha )}f\left(\gamma \right)g\left(\gamma^{-1}\circ \alpha \right)$. Hence, we may consider the von Neumann algebra ν(G) as the completion of the ∗-algebra $\left(C_{c}\left(G\right),⋆\right)$ with respect to the weak or strong topology on $B(L^{2}\left(G\right))$. Indeed, von Neumann’s theorem states that the closure of $R(C_{c}\left(G\right))$ in both the (ultra) weak topology and strong topology coincide and they are equal to its double commutant.

Now, we will take advantage of that C⊂G, then we may embed $L^{2}\left(C\right)$ as a closed subspace $V_{C}\subset L^{2}\left(G\right)$, extending trivially any function in $L^{2}\left(C\right)$, i.e., given $\psi \in L^{2}\left(C\right)$, we define Ψ(α)=ψ(α) if α∈C, and zero otherwise (note that we can do this because the space G is discrete countable equipped with the counting measure). Then, we can extend the right regular representation of the algebra C[C] in $B(L^{2}\left(C\right))$, to a representation on the larger algebra of bounded operators $B(L^{2}\left(G\right))$, by simply stating

$$\left(R\left(a\right)\Phi \right)\left(\beta \right)=\sum_{\left(\beta ,\alpha \right)\in C^{\left(2\right)}}a_{\alpha }\Phi \left(\beta \circ \alpha \right)\;,\;\forall \Phi \in L^{2}\left(G\right)\;.$$

Then, we see immediately that R(a) leaves the subspace $V_{C}=\{\Psi \in L^{2}\left(G\right)|\psi \in L^{2}\left(C\right)$ invariant. Using the same idea, the algebra of bounded operators in $L^{2}\left(C\right)$ can be identified with a subalgebra of the algebra of bounded operators in $L^{2}\left(B\right)$. In fact, if we decompose the Hilbert space $L^{2}\left(G\right)=V_{C}⊕V_{C}^{⊥}$, then any element *A* in the algebra $B(L^{2}\left(G\right))$ has a block decomposition given by

$$A=\left[\begin{array}{cc} A_{11} & A_{12}\\ A_{21} & A_{22} \end{array}\right]\;,\;A_{11}:V_{C}\to V_{C}\;,\;\;\;A_{12}:V_{C}\to V_{C}^{⊥},\;\;A_{21}:V_{C}^{⊥}\to V_{C},\;\;A_{22}:V_{C}^{⊥}\to V_{C}^{⊥}\;,$$

and the natural embedding $B\left(L^{2}\left(C\right)\right)\subset B\left(L^{2}\left(G\right)\right)$ is provided by identifying $B(L^{2}\left(C\right))$ with the subalgebra $B_{11}\left(L^{2}\left(G\right)\right)$ of $B(L^{2}\left(G\right))$ whose elements *A* have zero components $A_{ij}$, except the component $A_{11}$.

It is a simple computation to show that the double commutant $\nu \left(C\right)={\left(R\left(C\left[C\right]\right)\right)}^{\prime \prime }$ in the algebra $B(L^{2}\left(C\right))$ coincides with the double commutant of $R\left(C\left[C\right]\right)\subset B\left(L^{2}\left(C\right)\right)\subset B\left(L^{2}\left(G\right)\right)$, as a subset of the algebra $B(L^{2}\left(G\right))$, and because $B(L^{2}\left(C\right))$ is obviously weakly closed as a subalgebra of $B(L^{2}\left(G\right))$, then ν(C) is weakly closed in $B(L^{2}\left(C\right))$ (hence in $B(L^{2}\left(G\right))$). (Alternatively, ν(C) could have been defined as the weakly closure in $B(L^{2}\left(G\right))$ of R(C[C])).

Let us notice that if C⊂G is a causal structure in the groupoid G⇉Ω, then the opposite category $C^{\mathrm{opp}}$ can be identified with the inverse $C^{-1}$, of C in the groupoid G. Then, it is easy to check that $\nu \left(C^{-1}\right)=\nu {\left(C\right)}^{*}$, with $A^{*}$ denoting the ∗-operation on the von Neumann algebra ν(G) (which is just the induced adjoint operation from the ambient algebra $B(L^{2}\left(G\right))$).

### 4.2. Causal Structures in Groupoids and Triangular Operator Algebras

Because of the discussion at the end of the previous section, we conclude that the incidence algebra ν(C) of a causal category C in a groupoid G determines a closed subalgebra of the von Neumann algebra ν(G) of the groupoid. In addition, because $\Pi \left(C\cap C^{-1}\right)\subset i\left(\Omega \right)$, it follows that (in the discrete case again) we have an induced homomorphism of algebras: $\Pi_{*}:C\left[C\cap C^{-1}\right]=C\left[C\right]\cap C\left[C^{-1}\right]\to C\left[\Omega \right]$, given by

$$\Pi_{*}\left(a\right)=\sum_{\alpha \in C\cap C^{-1}}a_{\alpha }\Pi \left(\alpha \right)\;,$$

and, taking double commutants (or weak closures), we obtain a homomorphism of algebras $\Pi_{*}:\nu \left(C\right)\cap \nu {\left(C\right)}^{*}\to L^{\infty }\left(\Omega \right)$, as the double commutant of the Abelian algebra C[Ω] is just $L^{\infty }\left(\Omega \right)$.

Now, this situation is closely related to that of the so-called triangular operator algebras [33], that is, given a von Neumann algebra M, a closed subalgegra T⊂M is triangular with respect to M if $T\cap T^{*}$ is a maximal Abelian subalgebra (MASA) of M. In our current circumstances, M would be the von Neumann algebra ν(G) of the groupoid G, and the incidence algebra ν(C) would play the role of the triangular algebra T. Nevertheless, the situation we are dealing with is more general, as it is only the projection of $\nu \left(C\right)\cap \nu {\left(C\right)}^{*}$ under the canonical homomorphism Π=(t, s) that is a (not necessarily maximal) Abelian subalgebra (Such algebras studied in relation with Kadison–Singer algebras, see, for instance [32]). Strict causal categories on G will correspond to triangular algebras, such as in case we have $C\cap C^{-1}=i\left(\Omega \right)$ (recall Def. 3), and $\nu \left(C\right)\cap \nu {\left(C\right)}^{*}=L^{\infty }\left(\Omega \right)$. Moreover, of particular interest is the situation where M=B(H) is a type I factor. In such case, the maximal Abelian algebras A of type I factors fall into one of the following three cases [45,46,47]:

1. Diffuse case. A is isomorphic to $L^{\infty }\left(\Omega \right)$.
2. Discrete case. A is isomorphic to a diagonal algebra D of a matrix algebra.
3. Mixed case. A is isomorphic to $D⊕L^{\infty }\left(\Omega \right)$.

Hence, in the particular instance of a causal structure on the groupoids of pairs G=P(Ω) (hence, necessarily strict), the von Neumann algebra of the groupoid P(Ω) is isomorphic to the type I factor $B(L^{2}\left(\Omega \right))$. Consequently, the incidence algebra ν(C) is a triangular algebra with respect to the factor $\nu \left(P\left(\Omega \right)\right)=B\left(L^{2}\left(\Omega \right)\right)$, and the intersection $\nu \left(C\right)\cap \nu {\left(C\right)}^{*}=L^{\infty }\left(\Omega \right)$ is a maximal Abelian subalgebra of $B(L^{2}\left(\Omega \right)$. Thus, we have proved the following theorem.

**Theorem** **3.** *Let C be a strict causal category on the discrete countable groupoid G⇉Ω, then the incidence algebra ν(C) is a closed subalgebra of the von Neumann algebra ν(G) and it is a triangular operator algebra.*

We will conclude this section by proving a reconstruction theorem for strict causal structures on groupoids that answers the following question: Given a triangular operator subalgebra C⊂ν(G), does it define a causal structure on G? The answer, at least for discrete countable groupoids, is affirmative.

**Theorem** **4.** Let G⇉Ω be a discrete countable groupoid and C⊂ν(G) a triangular operator algebra with respect to the von Neumann algebra ν(G) such that $C\cap C^{*}$ is the maximal Abelian subalgebra $L^{\infty }\left(\Omega \right)$, then there is a one-to-one correspondence between weakly closed maximal indecomposable ideals J of C and outcomes x in Ω. Moreover, the relation x⪯y, defined by $J_{y}J_{x}\neq 0$, defines a causal set structure on Ω*, where $J_{x}$ is the ideal associated with x.*

**Proof.**  The proof of the theorem relies on the characterization of ultraweakly closed ideals I⊲M of a von Neumann algebra. In fact, there is a one-to-one correspondence, order preserving, between ultraweakly closed ideals *I* of M⊂B(H), and closed subspaces W⊂H such that the projection $p_{W}=p_{W}^{*}=p_{W}^{2}$ is in the center of M, and the ideal associated with *W* is $I_{W}=Mp_{W}$.

Hence, let us consider a weakly closed left ideal J⊲C and let *I* be the ideal in ν(G) generated by $J\cup J^{*}$. Then, the ideal *I* is associated with a closed subspace $W\subset L^{2}\left(G\right)$, $I=\nu \left(G\right)p_{W}$, and $p_{W}$ is the corresponding orthogonal projection. We define the subspaces $V_{C}$, $V_{C^{*}}$, as the closure of the action of the algebras *C* and $C^{*}$ on $L^{2}\left(G\right)$, respectively, i.e., $V_{C}=\bar{CL^{2}\left(G\right)}$, $V_{C^{*}}=\bar{C^{*}L^{2}\left(G\right)}$. Then, we consider the closed subspace $W\cap V_{C}$ and its associated orthogonal projection, denoted by $p_{C}$. The projection $p_{C}=p_{C}^{*}=p_{C}^{2}$ defines an ideal $I_{C}\subset I$ on ν(G) and, in addition, a left ideal on *C* given by $J_{C}=Cp_{C}$ because $p_{C}$ belongs to *C*.

Note that the ideal $J_{C}$ will be indecomposable in *C* only if $p_{C}$ is such that the subspace $W\cap V_{C}$ is one-dimensional generated by an element α∈G, i.e., $W\cap V_{C}=C|\alpha \rangle$, in such a case, we will denote the projection $p_{C}$ as $p_{\alpha }$ and the ideal as $J_{\alpha }$. However, if $C\cap C^{*}\subset L^{\infty }\left(\Omega \right)$, then $J_{\alpha }\cap J_{\alpha }^{*}\subset L^{\infty }\left(\Omega \right)$, then $p_{\alpha }$ is a projection on $L^{\infty }\left(\Omega \right)$ which can only occur if α∈Ω, i.e., $\alpha =1_{x}$ for some x∈Ω. Hence, the ideal $J_{C}$ is the left ideal in *C* generated by an outcome x∈Ω and subsequently denoted as $J_{x}$.

On the other hand, $W\cap V_{C}$ contains the span of the action of J on $L^{2}\left(G\right)$, hence $J\subset J_{x}$, but if J is maximal, then it must be $J_{x}$. □

Theorems 1 and 2 are immediate corollaries of the previous result when *C* is the incidence algebra of a strict causal category C⊂G.

Note that if the groupoid G is a countable group Γ, then the previous theorem applies to the situation that C⊂ν(Γ) is a closed subalgebra of the von Neumann algebra of the group and $C\cap C^{-1}=CI$, in which case we conclude that there are no nontrivial indecomposable ideals of *C*.

## 5. Conclusions and Discussion

A novel approach to causality in the context of category theory and groupoids is introduced. Causal relations on sets Ω are associated with the choice of a category C over Ω satisfying some natural properties. They reproduce the vast majority of various approaches to causality introduced before: from Einstein’s geometric causality to Sorkin’s causal sets theories. In addition, they will allow to place them in the context of quantum mechanical systems by using their Schwinger’s inspired groupoidal picture. In fact, a causal structure on a groupoid is just a causal category that is a subcategory of the groupoid. The analytical viewpoint provided by the von Neumann algebra of the groupoid allows to deal with causal relations from the point of view of their associated algebras. In this setting, it is found that strict causal categories determine triangular operator algebras and that, in the particular instance of discrete countable groupoids, it is found that such algebras determine a causal set structure on the space Ω, providing not only a new proof, but a significant extension of Sorkin’s theorem on the characterization of causal structures by means of their incidence algebra and its class of ideals. Particular instances of this theorem will be discussed elsewhere, most significantly countably non-locally finite digraphs.

There remains, though, many relevant questions concerning the interplay of causality and quantum mechanics that require further analysis and development. Most important among them is the problem of time in quantum mechanics discussed at the beginning of Section 4. In addition to this, the treatment of relativistic covariant systems from the perspective discussed in this paper is a significant problem that will be dealt with in further publications.

From a purely mathematical standpoint, apart from various problems that have been already pointed out in the main text, such as the extension of the characterization of causal structures to nondiscrete groupoids or the relation between nonstrict causal categories and general Kadison–Singer algebras, the relation between causal structures, Kadison–Singer algebras, and von Neumann algebras associated with groupoids is a new and promising path of research that will be pursued by relating it to previous attempts to tamper the problem of causality in quantum systems by using noncommutative geometrical ideas (see, for instance, [48,49,50]).

Finally, we expect that the use of the ideas presented in a rather embryonic form in the present paper will help to offer a new path towards a proof of the CPT theorem [51] and the spin–statistics theorem without relying on covariant quantum field theoretical arguments, a problem that goes back to W. Pauli and for which G. Sudarshan offered a solution partly using Schwinger’s formalism (see [52] for a comprehensive description).

## Data Availability

Not applicable.
